# Exhaled Volatile Organic Compounds for Identifying Patients With Chronic Pulmonary Aspergillosis

**DOI:** 10.3389/fmed.2021.720119

**Published:** 2021-09-23

**Authors:** Zheng-Tu Li, Pei-Ying Zeng, Zhao-Ming Chen, Wei-Jie Guan, Tong Wang, Ye Lin, Shao-Qiang Li, Zhi-Juan Zhang, Yang-Qing Zhan, Ming-Die Wang, Guo-Bin Tan, Xue Li, Feng Ye

**Affiliations:** ^1^State Key Laboratory of Respiratory Disease, National Clinical Research Center for Respiratory Disease, National Center for Respiratory Medicine, Guangzhou Institute of Respiratory Health, The First Affiliated Hospital of Guangzhou Medical University, Guangzhou, China; ^2^Department of Thoracic Surgery, Guangzhou Institute for Respiratory Health, The First Affiliated Hospital of Guangzhou Medical University, Guangzhou, Guangdong, China; ^3^Institute of Mass Spectrometry and Atmospheric Environment, Jinan University, Guangzhou, China; ^4^Guangdong Provincial Engineering Research Center for On-Line Source Apportionment System of Air Pollution, Guangzhou, China; ^5^College of Pharmacy, Hena University of Chinese Medicine, Zhengzhou, China; ^6^Guangzhou Hexin Instrument Co., Ltd., Guangzhou, China

**Keywords:** chronic pulmonary aspergillosis, volatile organic compounds, metabolomics, exhalation, single-photon ionization-mass spectrometry

## Abstract

**Background:** Diagnosing chronic pulmonary aspergillosis is a major challenge in clinical practice. The development and validation of a novel, sensitive and specific assay for diagnosing chronic pulmonary aspergillosis is urgently needed.

**Methods:** From April 2018 to June 2019, 53 patients with chronic pulmonary aspergillosis (CPA), 32 patients with community-acquired pneumonia (CAP) and 48 healthy controls were recruited from the First Affiliated Hospital of Guangzhou Medical University. Clinical characteristics and samples were collected at enrollment. All exhaled breath samples were analyzed offline using thermal desorption single-photon ionization time-of-flight mass spectrometry; to analyze the metabolic pathways of the characteristic volatile organic compounds, serum samples were subjected to ultrahigh-performance liquid chromatography.

**Results:** We identified characteristic volatile organic compounds in patients with chronic pulmonary aspergillosis, which mainly consisted of phenol, neopentyl alcohol, toluene, limonene and ethylbenzene. These compounds were assessed using a logistic regression model. The sensitivity and specificity were 95.8 and 96.9% for discriminating patients in the CPA group from those in the CAP group and 95.8 and 97.9% for discriminating patients in the CPA group from healthy controls, respectively. The concentration of limonene (*m/z* 136) correlated significantly positively with anti-*Aspergillus fumigatus* IgG antibody titers (*r* = 0.420, *P* < 0.01). After antifungal treatment, serum IgG and the concentration of limonene (*m/z* 136) decreased in the subgroup of patients with chronic pulmonary aspergillosis.

**Conclusions:** We identified VOCs that can be used as biomarkers for differential diagnosis and therapeutic response prediction in patients with chronic pulmonary aspergillosis.

## Importance

In this study, it is promising to apply TD-SPI-TOF-MS to quickly evaluate the possibility of using breath VOCs to fast screen chronic pulmonary aspergillosis (CPA) patients. And several characteristic volatile organic compounds were identified as potential biomarkers for differential diagnosis and therapeutic response prediction in patients with CPA. Furthermore, the serum metabolism also used to preliminarily explore the source of exhaled VOCs. The findings of our study will provide a new, relatively non-invasive and easy to operate diagnostic method for the future clinical diagnosis of CPA.

## Introduction

Pulmonary aspergillosis is common in immunosuppressed patients and a major global health burden, affecting more than 3 million people worldwide (1, 2). Patients with chronic pulmonary aspergillosis (CPA) frequently have comorbidities and atypical clinical symptoms, making early diagnosis and treatment difficult. To date, the diagnosis of CPA mainly relies on pathology, serology and imaging. However, these diagnostic methods are time consuming and may be invasive, and personnel performing microscopy analyses need to be professional and experienced; therefore, missed or delayed diagnosis occurs in a considerable proportion of patients, which might increase the mortality rate (3, 4).

Recent scientific advances have allowed for the rapid, non-invasive, and convenient diagnosis of respiratory disease with cutting-edge technologies (5). Measuring the patterns of volatile organic compounds (VOCs) in exhaled breath is a novel non-invasive metabolomic approach for studying the molecular signatures of respiratory disease (6–10), and gas chromatography-mass spectrometry (GC-MS) has identified several characteristic VOCs in patients with pulmonary aspergillosis (11–13). Nonetheless, breath analysis of pulmonary aspergillosis is mostly based on *Aspergillus* strains *in vitro*, and there are a limited number of volatile substances that can be detected by GC-MS. Thermal desorption—single-photon ionization—mass spectrometry (TD-SPI-MS) has a high sensitivity, is user-friendly, and can be applied to detect VOCs with a low concentration or a wide range of substances (9, 14). In general, metabolomic analysis can quantitatively analyze all metabolites, which helps in evaluating associations between metabolites and pathophysiological changes (15). Indeed, a number of studies have reported that serum metabolites can be used as biomarkers of infectious diseases for diagnosing and evaluating clinical severity (16–19).

In this study, we aimed to perform broad-spectrum screening of exhaled VOCs and metabolomic analysis by applying TD-SPI-MS for patients with CPA. The metabolic pathways of such metabolites may further reveal the components and biological roles of the characteristic VOCs in exhaled breath associated with pulmonary aspergillosis. Our findings provide a novel tool for the non-invasive, simple and reliable clinical diagnosis of CPA.

## Methods

### Patients

Between April 2018 and June 2019, patients with CPA, patients with community-acquired pneumonia (CAP) and healthy controls were recruited from the First Affiliated Hospital of Guangzhou Medical University. Patients with CPA according to the guidelines of diagnosis and management of *Aspergillus* diseases were enrolled (group CPA) (20). The diagnosis of CAP was made according to the guidelines of diagnosis and treatment of CAP (group CAP) (21). The healthy controls did not have any documented pulmonary infectious, interstitial or malignant lung diseases (group N). We excluded patients who were younger than 18 years old, declined exhaled breath analysis, had ingested foods or brushed their teeth after 22:00 p.m. the night before sample collection or were currently smokers or had consumed alcohol within 2 months prior to the examination.

The guidelines (20) for the diagnosis and treatment of pulmonary aspergillosis recommend at least 6 months of voriconazole therapy (200 mg every 12 h) for CPA. As the plasma concentration of voriconazole was detected to maintain the recommended effective concentration range (1~5.5 mg/L), four time points were scheduled for follow-up during the antifungal treatment. The patients were followed up for 9 months (before treatment and at 3, 6, and 9 months after treatment).

### VOC Analysis

TD-SPI-MS was performed to obtain qualitative and quantitative data. See Supplementary Material for the detailed methods and precautions of exhaled breath collection.

### Serum Metabolite Analysis and Metabolic Pathways

Patients fasted for 8–10 h before exhaled gas collection, followed by venipuncture (4 ml) in the morning to collect peripheral blood. The samples were centrifuged at 3,000 rpm for 15 min, and the serum was stored at −80°C for subsequent analysis. For each assay, 100 μl serum plus 300 μl methanol and 20 μl internal standard were added to a centrifuge tube. After vortexing for 30 s, the samples were subjected to ultrasonic treatment in an ice bath for 5 min and kept at −20°C for 2 h. After centrifugation at 13,000 rpm at 4°C for 15 min, 200 μl of the supernatant was transferred to a 2-ml sample vial. All serum metabolites were analyzed using an ultrahigh-performance liquid chromatography-pristine instrument.

### Statistical Analysis

Clinical data were analyzed using IBM SPSS Statistics (version 20, SPSS Inc., USA). A *P*-value of 0.05 or less indicated statistical significance. Categorical data are presented as numbers and percentages and continuous data as medians and ranges or interquartile ranges (IQRs). Comparison of continuous data was performed using Student's *t*-test or Mann-Whitney test. Fisher's exact test was applied to compare categorical variables. Partial least squares discrimination analysis (OPLS-DA) with SIMCA software (version 14.1, MKS Umetrics, Sweden) (22) was adopted. High-dimensional data were further processed through dimensionality reduction, and the variable importance for projection (VIP) in the OPLS-DA model was calculated (23). For VIPs equal to or >1.0, an array of characteristic ions in the CPA group was selected as potential targets. As each substance may have the same germplasm charge ratio, each *m/z* value can correspond to one or more VOCs. The possible ion range can be inferred according to the CAS (Chemical Abstracts Service, USA).

Metabolites were qualitatively evaluated according to the KEGG (Kyoto Encyclopedia of Genes and Genomes) (http://www.genome.jp/kegg/) database and a customized database. Multivariate statistical analysis was performed using the normalized data matrix with PCA, PLS-DA, OPLS-DA and sequencing tests. Multivariate (OPLS-DA) and univariate statistical (Student's *t*-test) analyses were applied to screen and cluster differentially expressed metabolites, perform metabolic pathway KEGG enrichment analysis, and construct a metabolite regulation network. Potential metabolic pathways between exhaled breath and blood were explored by performing Venn analysis and enrichment analysis. The digital matrix composed of the selected characteristic VOCs was imported into MATLAB (version R2017b, MathWorks, Inc., USA) to construct different classification models, and the accuracy, sensitivity and characteristics of the models were verified by binary logistic regression analysis. The optimal model was selected for simulation operation, which further confirmed the value of characteristic VOCs for CPA diagnosis. Pearson's correlation analysis was used to analyze correlations between exhaled breath VOC expression levels and anti-*Aspergillus* IgG titers in the CPA group.

## Results

### Baseline Characteristics

We included 53 patients in the CPA group and 32 in the CAP group and 48 subjects in group N. The study flow chart is provided in Supplementary Figure 1, and the baseline characteristics of the included participants are shown in Table 1. Most of the subjects were male (59.4%), and the male subjects were significantly older than the female subjects [median: 54 (range 38–65 years) vs. 39 (range 29–54 years)]. Patients in the CPA group rarely smoked or drank alcohol. Additionally, age and sex differed significantly between the CPA group and group N (*P* < 0.01). Both weight and smoking history (*P* < 0.01), but not height, drinking history or eating habits, differed significantly among the three groups.

**Table 1:** Baseline characteristics of the study participants.

**Characteristics**	**Group CPA**	**Group CAP**	**Group N**	***P*-value**
	**(*n* = 53)**	**(*n* = 32)**	**(*n* = 48)**	
Age (year, x ± S)	49.0 ± 15.8	38.0 ± 18.5	26.2 ± 7.0	0.001
Sex, male (No, %)	35 (66.0%)	11 (34.4%)	33 (68.8%)	0.004
Height (cm, x ± S)	162.9 ± 10.0	161.4 ± 9.0	162.2 ± 9.2	0.762
Weight (kg, x ± S)	51.6 ± 10.9	56.3 ± 12.5	59.0 ± 10.6	0.003
**Smoking status** ^**a**^				
Ex-smoker (No, %)	13 (24.5%)	5 (15.6%)	3 (6.3%)	0.010
Never-smoker (No, %)	40 (75.5%)	27 (84.4%)	45 (93.8%)	
**Drinking status** ^**a**^				
Ex-drinker (No, %)	3 (5.8%)	1 (3.1%)	0	0.121
Never-drinker (No, %)	50 (94.3%)	31 (96.9%)	48 (100.0%)	
**Eating habits** ^**a**^				
Light diet (No, %)	45 (83.2%)	30 (93.8%)	43 (89.6%)	0.078
Non-light diet (No, %)	8 (16.8%)	2 (6.3%)	5 (10.4%)	

*a: Ex-smokers were defined as those who smoked continuously for at least 6 months but had quit smoking for more than 2 months at the time of recruitment; the definitions of ex-drinkers were similar to those of ex-smokers; light diet refers to daily diet habits, including spicy food such as pepper, garlic, and green onion*.

### Characteristic Exhaled VOCs

To avoid artifacts, the sampling temperature was controlled between 20 and 25°C, and a regular ultraviolet lamp was applied for environmental irradiation to minimize contamination before sample collection. Despite certain differences in demographic characteristics, we recruited study participants without a recent smoking history.

**Figure 1:**
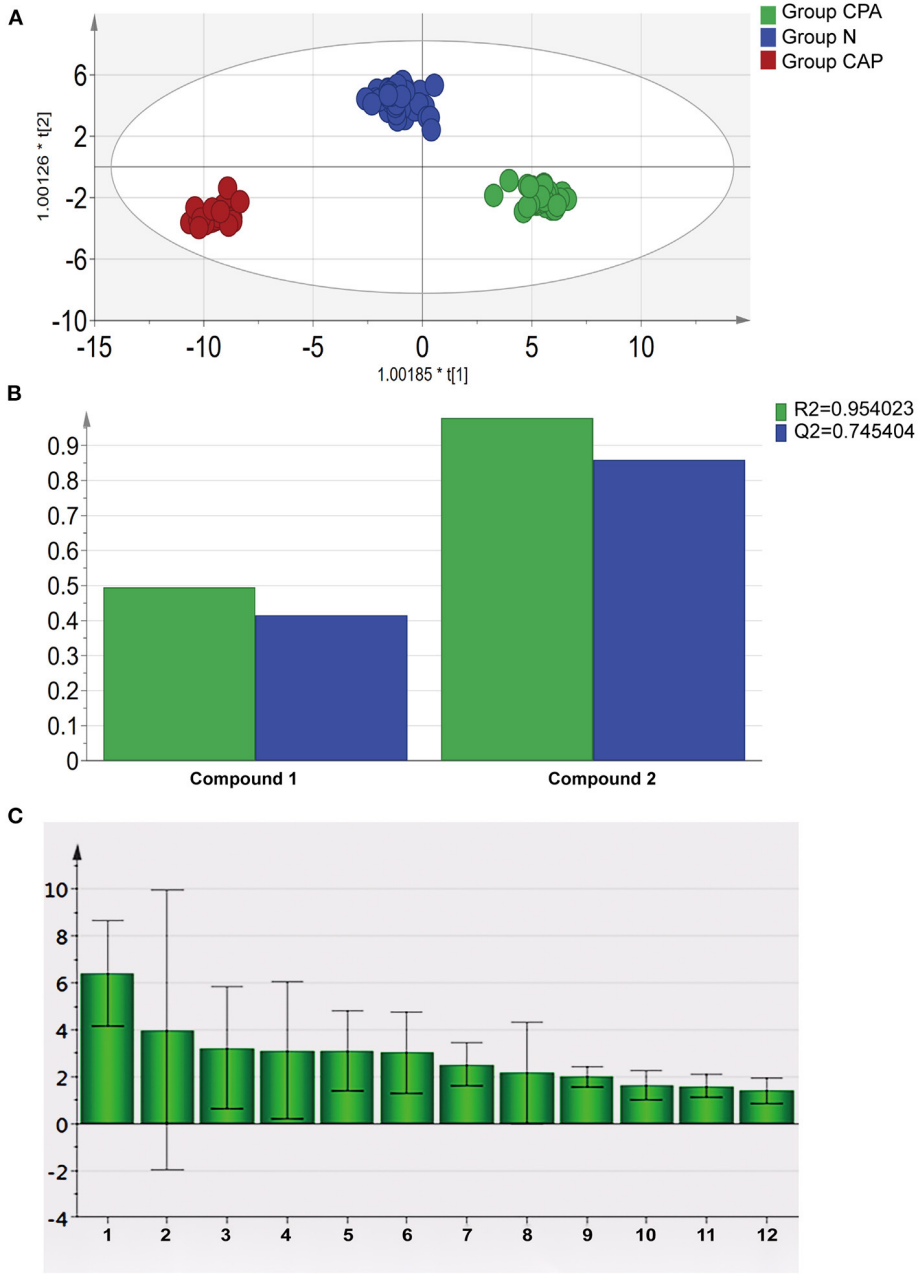
Statistical analysis of characteristic VOCs in exhaled breath of patients with CPA. **(A)** OPLS-DA score plot of characteristic VOCs in exhaled breath samples in group CPA, group CAP and group N. **(B)** Parameters of the OPLS-DA model of characteristic VOCs in exhaled breath samples in group CPA, group CAP and group N. **(C)** VIP value of characteristic VOCs in exhaled breath samples of patients with CPA.

According to the OPLS-DA model of the entire cohort, VOCs in exhaled breath were capable of distinguishing the three groups (R^2^ = 0.954, Q^2^ = 0.745, Figures 1A,B). Therefore, the original data model explained the differences among the CPA, CAP and N groups. The *m/z* value of the characteristic VOCs was derived based on the VIP value (Figure 1C). After incorporating information from Chemical Abstracts Database (Chemical Abstracts Service, CAS), the characteristic VOCs in patients with CPA were found to be ketones, aldehydes, esters, hydrocarbons and benzenes; the detailed characteristics of these compounds are shown in Table 2.

**Table 2:** Reference compounds of characteristic VOCs of patients in the CPA group.

**Number**	**m/z value**	**Compound name**	**CAS number**
1	m/z 58	Propanone/Acetone	7-64-1
2	m/z 62	Dimethyl sulfide	75-18-3
		*Ethylthiol*	75-08-1
3	m/z 68	Isoprene/2-Methyl-1,3-Butadiene	78-79-5
4	m/z 69	1,2-Pentadiene	591-95-7
		Ethyl acetate	141-78-6
5	m/z 88	Neopentyl alcohol	75-84-3
		2-Methyl-2-butanol	75-85-5
6	m/z 92	Toluene	108-88-3
7	m/z 94	Phenol	108-95-2
		Methyl disulfide	624-92-0
8	m/z 106	M-Xylene	108-38-3
		P-Xylene	106-42-3
		Ethylbenzene	100-41-4
9	m/z 134	1-Methyl-2-propylbenzene	1074-17-5
		1-Methyl-4-propylbenzene	1074-55-1
10	m/z 136	2-Methyl-3-propylpyrazine	15986-80-8
		3,7-dimethyl-1,3,7-Octatriene	502-99-8
		2-(sec-Butyl)pyrazine	124070-52
		Dipentene	138-86-3
		Phenyl acetate	122-79-2
11	m/z 281	Diphenyl hydrogen phosphate	838-85-7
12	m/z 282	—	—

### Characteristic Serum Metabolites and Metabolic Pathways

At present, existing theories show that the exhaled VOCs are the product of blood metabolism after pathogen infection (5, 6, 24). Therefore, the purpose of our study on serum metabolomics is to preliminarily explore the source of exhaled VOCs. To identify characteristic serum metabolites, we randomly selected samples from subjects with paired serum samples (10 cases in group CPA, 5 in group CAP and 5 in group N). Except for a significant difference in age between group CPA and group N, there were no significant differences in sex, weight or height among the three groups (*P* > 0.05).

**Figure 2:**
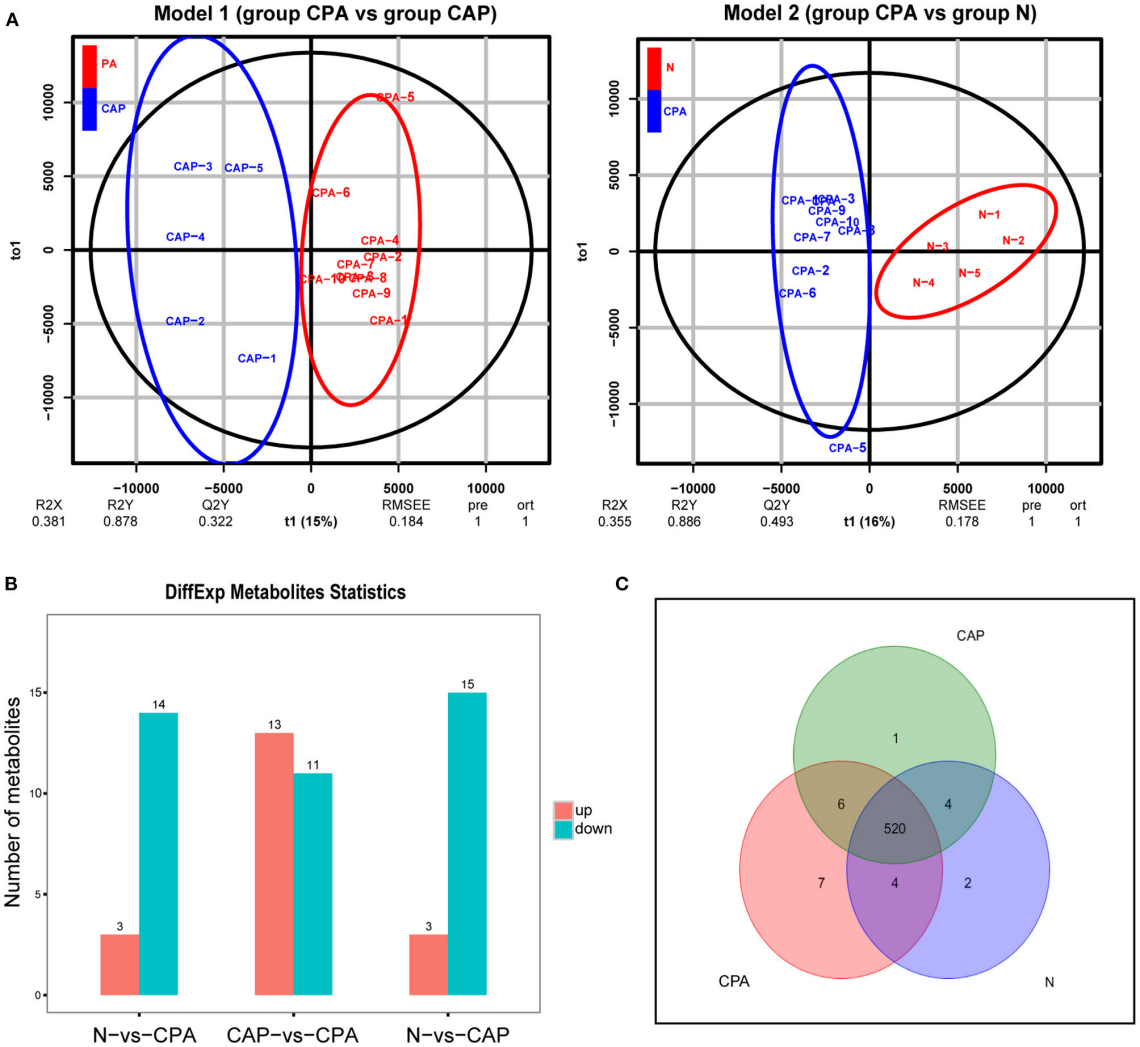
Serum metabolome analysis for distinguishing group CPA from group CAP and group N. **(A)** OPLS-DA score plot of serum characteristic metabolites between the CPA group and the CAP and N groups. **(B)** Quantity change of different serum metabolites among group CPA, group CAP and group N. **(C)** Venn analysis of serum characteristic metabolites in patients with CPA.

The OPLS-DA score chart (Figure 2A) showed that the original model was able to explain the differences in serum metabolites among the CPA, CAP and N groups. According to the VIP value of OPLS-DA and between-group comparisons (Student's *t*-test), we identified 24 differentially expressed metabolites between groups CPA and N and 17 differentially expressed metabolites between groups CPA and CAP (Figure 2B). A Venn diagram further illustrated 7 metabolites in group CPA that were differentially expressed compared with groups N and CAP (Figure 2C).

**Figure 3:**
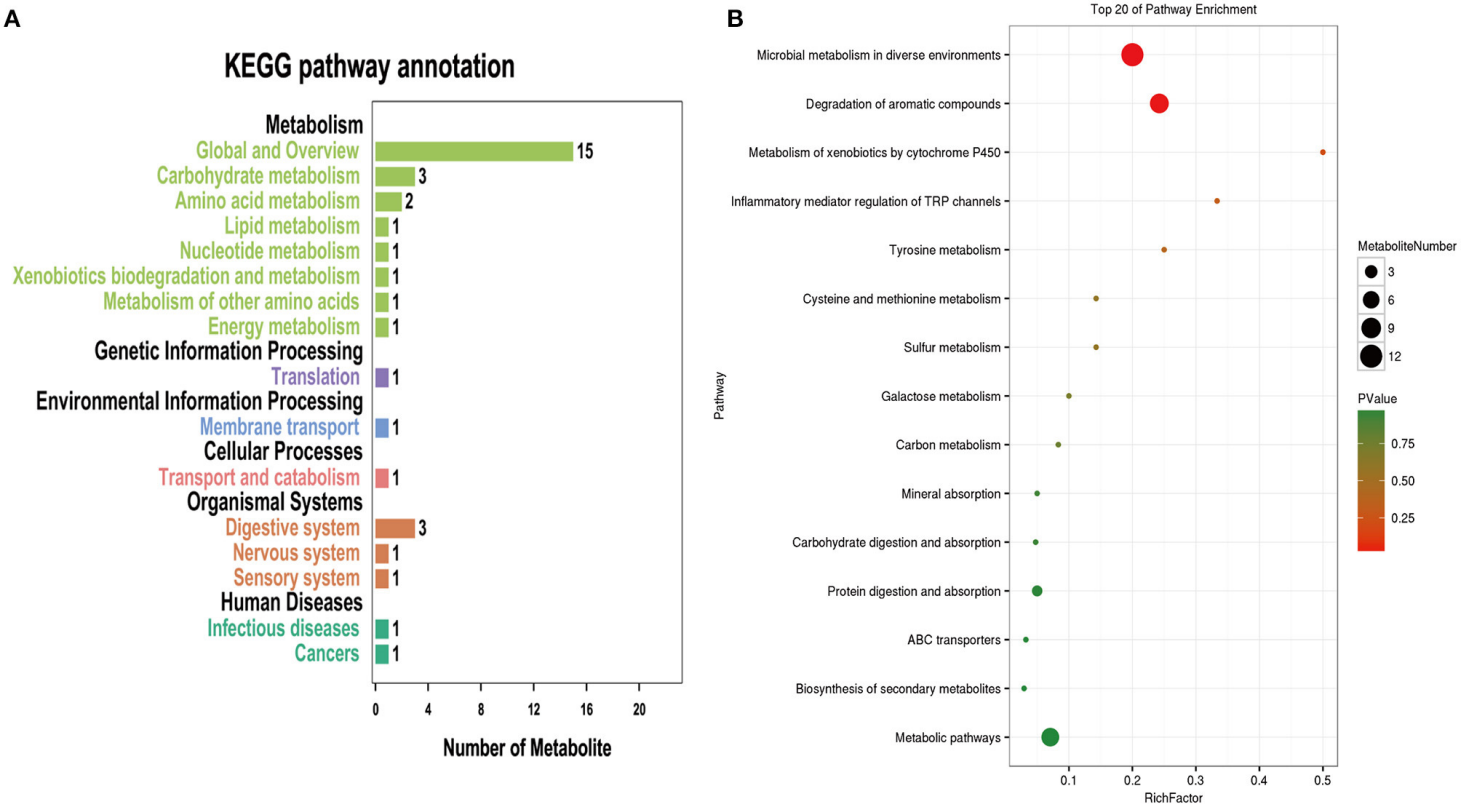
Common metabolic pathways of characteristic VOCs and serum-specific metabolites in patients with CPA. **(A)** Pathway enrichment histogram of candidate metabolites. **(B)** Bubble diagram of pathway enrichment results of candidate metabolites. the P*value ranges from 0 to 1. The closer to zero, the closer the color is to red, indicating that the enrichment is more significant.

**Figure 4:**
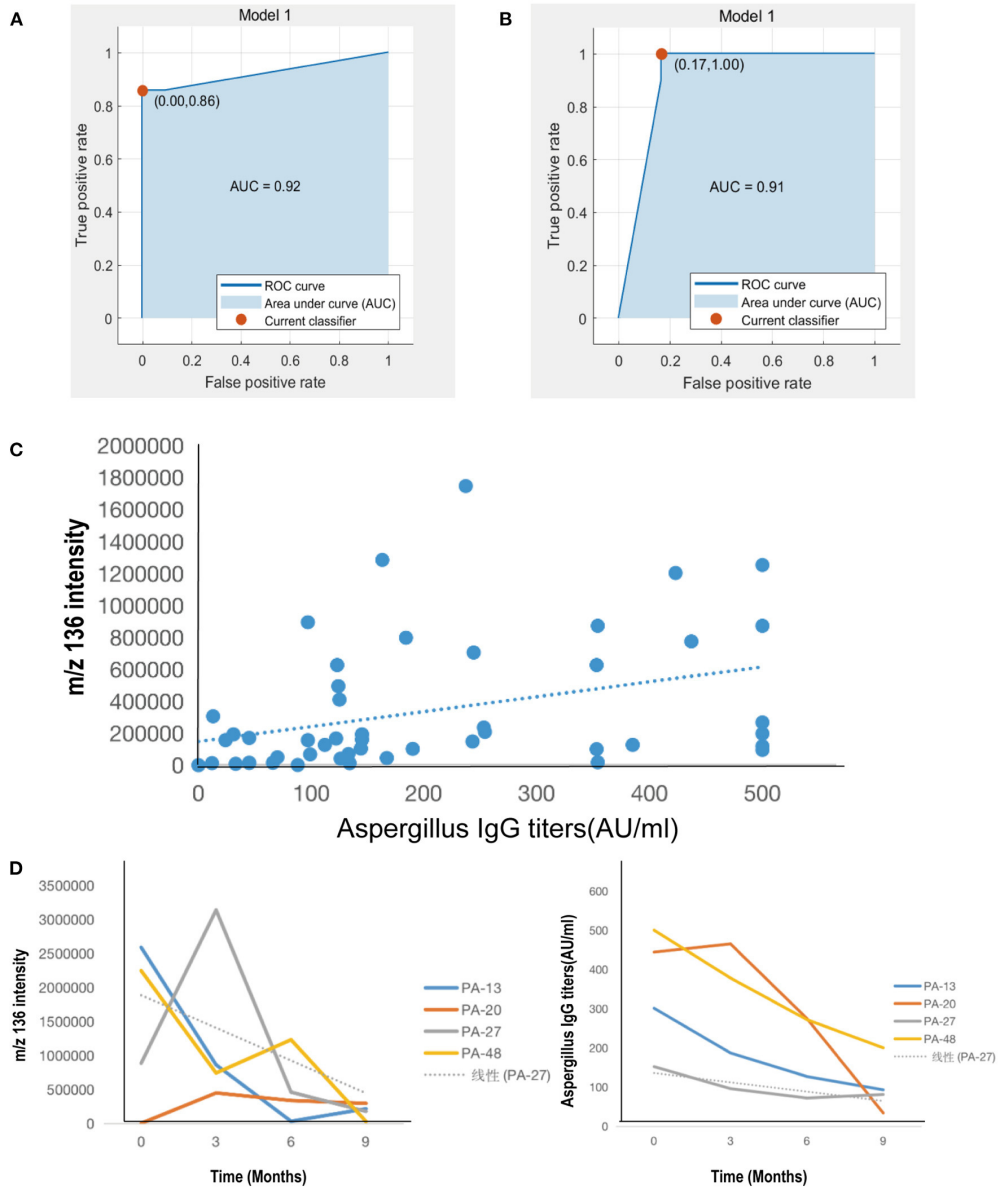
Clinical correlation of characteristic VOCs in exhaled breath of patients with CPA. **(A)** Univariate logistic regression model of the CPA group and CAP group. **(B)** Univariate logistic regression model of group CPA and group N. **(C)** Correlation analysis between characteristic VOCs and serum anti-*Aspergillus fumigatus* IgG antibody titers. **(D)** Dynamic changes in levels of breath characteristic VOCs and serum anti-*Aspergillus fumigatus* IgG antibody titers of 4 patients in the CPA group for baseline and follow-up in the third, sixth, and ninth month after voriconazole treatment.

The same characteristic VOC expressed metabolites between exhaled breath and serum in CPA patients were enriched in different metabolic pathways to clarify the source. Pathway analysis based on the latest KEGG database for differentially expressed metabolites was assigned to six level-1 KEGG pathways (Figure 3A). Pathway enrichment analysis identified the most significantly altered metabolic pathways related to microbial metabolism in diverse environments (pathway 1, *P* < 0.05) and degradation of aromatic compounds (pathway 2, *P* < 0.05) (Figure 3B). In these metabolic pathways, the expression profiles of the characteristic VOCs (including phenol, neopentyl alcohol, toluene, limonene and ethylbenzene) between exhaled breath and serum matched (Table 3). These compounds were tested by a logistic regression model, with a sensitivity and specificity of 95.8 and 96.9% for discriminating patients in the CPA group from those in the CAP group and 95.8 and 97.9% for discriminating patients in the CPA group from healthy controls, respectively (Figures 4A,B).

**Table 3:** Common metabolic pathways of characteristic VOCs in exhaled breath and serum metabolites of patients in the CPA group.

**KEGG pathway ID**	**Pathway**	***P*-value**	Pathway C-id**	**Compound**
ko01120	Microbial metabolism in diverse environments	0.0068	C00146	Phenol
			C00556	Neopentyl alcohol*
			C00556	Neopentyl alcohol
			C01455	Toluene
			C06104	Dipentene*
			C07111	Ethylbenzene
ko01220	Degradation of aromatic compounds	0.0140	C00146	Phenol
			C00556	Neopentyl alcohol*
			C01455	Toluene
			C06104	Di pentene*
			C07111	Ethylbenzene

1. **: ID (C number) of metabolites in the KEGG database; 2. *: serum differential metabolites; 3. Other unmarked metabolites are characteristic VOCs of exhaled breath.

### Correlation of Characteristic VOCs With Clinical Parameters

In the CAP group, 13 patients (40.6%) had *Mycoplasma* pneumonia, 11 (34.4%) bacterial pneumonia and 8 (25.0%) viral pneumonia. Among CPA patients, the main presenting symptoms were fever, cough, expectoration, hemoptysis and dyspnea. The dominant radiologic characteristics included cavitation, nodules, patchy shadows and ground-glass opacities. There were no significant differences in the neutrophil count or hemoglobin and procalcitonin levels (*P* < 0.05, Supplementary Table 1).

We next further explored the association between characteristic VOCs in exhaled breath and anti-*Aspergillus fumigatus* IgG antibody titers in the serum of those in the CPA group. Although the concentration of characteristic VOCs (neopentyl alcohol, phenol, toluene, ethylbenzene) did not correlate significantly with serum IgG antibody titers (*P* > 0.05), the limonene concentration did correlate positively (*r* = 0.420, *P* < 0.01, Figure 4B).

We finally evaluated the association between dynamic changes in characteristic VOC expression levels and disease outcomes as well as the efficacy of antifungal treatment. Data were available during longitudinal follow-up four patients (Figure 4D). The concentration of VOC *m/z* 136 (limonene) progressively declined after the initiation of antifungal treatment. After anti-fungal treatment for 9 months, the health status of three patients (PA-13, PA-27 and PA-48) improved, as reflected by the alleviation of cough or hemoptysis, decreased anti-*A. fumigatus* IgG antibody titers (negative in one case, positive in two cases), decreased absorption of lesions on chest computed tomography, and a stable blood voriconazole concentration. In support of these clinical signs, the concentration of exhaled breath limonene was also markedly decreased. After 3 months of treatment, PA-27 was readmitted to the hospital due to the recurrence of hemoptysis. Meanwhile, the anti-*Aspergillus* IgG antibody titer decreased compared with the first clinical visit. In contrast, the concentration of exhaled breath limonene increased significantly and then decreased significantly with improvement of the disease. Patient No. PA-20 exhibited disease progression after 9 months of antifungal treatment, with a recurrence of hemoptysis and an increase in pulmonary infiltration, as revealed by chest computed tomography. Nevertheless, negative conversion was achieved for anti-*Aspergillus* IgG antibodies, with the concentration of limonene remaining relatively stable over the course of follow-up (Figure 4C).

## Discussion

We identified several characteristic VOCs by performing TD-SPI-MS in the exhaled breath and serum of patients with CPA, revealing biological pathways associated with microbial metabolism in diverse environments and the degradation of aromatic compounds. Detection of these compounds can effectively distinguish patients with CPA; therefore, some characteristic compounds from exhaled breath or serum VOCs might help with the diagnosis of CPA.

Exhaled breath analysis is prone to bias by environmental or host factors (25, 26). In our study, we conducted rigorous screening for the inclusion of patients, environmental controls and repeated sample collection to minimize measurement bias. The statistical analysis model consistently indicated a good separation of exhaled breath and serum VOCs among the three groups. Our findings confirmed the results from the study by Koo et al. (12), in which alkenes, ketones and alkanes (including limonene) were detected by GC-MS in the exhaled breath of patients with pulmonary aspergillosis. These findings indicate the value of these compounds as exhaled biomarkers to differentiate pulmonary aspergillosis from other diseases and healthy subjects as well as alkenes and ketones as exhaled breath biomarkers (such as acetone, limonene, isoprene and 1,2-pentadiene). Benzene compounds have not been confirmed as characteristic VOCs of CPA. Our study found that phenol, toluene and ethylbenzene might serve as biomarkers for CPA. The high sensitivity and specificity for differentiating the CPA group might be related to differences in expression levels of benzene, toluene, xylene and chlorobenzene, which are oxidative stress markers of aspergillosis (27). For example, 2-pentyl furan has been considered an important marker of exhaled breath VOCs of patients with *Aspergillus* infection (11, 28, 29). *Aspergillus spp*. produce 2-PF on blood agar, nutrient agar and other media. In our study, the substance at *m/z* 128 had a VIP value of <1 and was not confirmed as a characteristic VOC of PA. 2-PF might have originated from hemoglobin, which is produced by blood agar culture medium or pulmonary hemorrhage rather than *Aspergillus spp. per se* (30). Therefore, it is important to explore the source of these compounds, for instance, by performing metabolomic analysis.

There are more than 200 metabolites related to *Aspergillus sp*. infections (31), most of which are toxins, yet most metabolites produced by interactions with the host are still unknown. In our study, the main metabolic pathways involved in the characteristic VOCs of exhaled breath and the differentially expressed metabolites in serum consisted of microbial metabolism compounds in diverse environments and the products of aromatic compound degradation. Characteristic VOCs in microbial metabolism and metabolic pathways (degradation of aromatic compounds) were shared between the exhaled breath and serum samples. The main metabolites (including phenol, neopentyl alcohol, toluene, limonene and ethylbenzene) suggested that *Aspergillus sp*. infection might result in the production of characteristic metabolites through microbial metabolism and aromatic compound degradation (12, 30). Consistent with this finding, our study also showed limonene to be a potential characteristic compound, thus confirming its value for differential diagnosis based on exhaled breath analysis. It has been reported that aromatic hydrocarbons can regulate inflammatory T cell and dendritic cell (DC) responses (32) and induce oxidative stress and airway tissue injury (33). These compounds in the exhaled breath might also be products of the host's immune response to pathogens (5).

We further confirmed the sensitivity and specificity of the abovementioned characteristic VOCs. According to a previous study, the sensitivity and specificity of exhaled breath VOCs were 95.8 and 96.9% for discriminating patients with CPA from those with CAP and 95.8 and 97.9% for discriminating patients with CPA from healthy controls, respectively. Compared with traditional serologic testing, such as GM tests using serum and bronchoalveolar lavage samples (34), VOC assays *via* TD-SPI-MS might better indicate candidate biomarkers in the exhaled breath of CPA patients. The current international guidelines suggest that IgG antibody detection is the most sensitive laboratory test for the diagnosis of chronic cavitary pulmonary aspergillosis (CCPA) (3). We also showed that changes in serum anti-*A. fumigatus* IgG antibody titers in patients with CPA before and after antifungal treatment may sensitively reveal the occurrence and progression of CPA. Anti-fungal therapy reportedly decreases levels of serum IgG titers in patients with CPA (35). Importantly, during a 9-month follow-up, the intensity of limonene also decreased significantly. In addition, there were two patients who experienced CPA recurrence. Among them, despite an apparent decrease in IgG titer, the limonene concentration remained relatively high, suggesting that exhaled VOCs might be used for evaluating the therapeutic response. Hence, the concentration of characteristic VOCs might indicate the efficacy of antifungal treatment.

Several limitations need to be acknowledged. First, interpretation of the findings (particularly the follow-up investigation) was limited by the small sample size, and only the conformed CPA patients, was taken as the study group. In the future study, we will recruit all suspected CAP patients as many as possible. Thus, there will be enough remaining suspected CAP patients which were excluded from the real CPA patients can be used as one of the control groups. Further investigations that take into account different subtypes of pulmonary aspergillosis are also necessary. Second, our study lacks an *in vitro* analysis of *Aspergillus* strains, which will help validate our findings of cell experimental results. Third, although the TD-SPI-MS instrument has a wide detection range and high sensitivity, the findings should be further validated by GC-MS and other mass spectrometry technologies because there is no unified detection standard for exhaled VOCs and detection results among instruments may differ. Finally, heterogeneity in the underlying diseases of the CAP group might have affected the discriminatory capacity of VOCs, though the inclusion of patients with CAP of different pathogens might have increased the generalizability of our findings.

## Conclusion

Exhaled breath VOCs (phenol, neopentyl alcohol, toluene, limonene and ethylbenzene), which may help effectively discriminate patients with CPA, are involved in endogenous metabolism. Limonene might be a novel biomarker that reflects the clinical course and treatment response of CPA.

## Data Availability Statement

The data-sets generated and/or analyzed during the current study are not publicly available due to the presence of sensitive (confidential) participants' information. Requests to access the datasets should be directed to tu276025@gird.cn.

## Ethics Statement

The studies involving human participants were reviewed and approved by the Ethics Committee of the First Affiliated Hospital of Guangzhou Medical University. The patients/participants provided their written informed consent to participate in this study. Written informed consent was obtained from the individual(s) for the publication of any potentially identifiable images or data included in this article.

## Author Contributions

Z-TL, P-YZ, XL, and FY conceived of and designed the study. Z-TL, P-YZ, Z-MC, W-JG, and FY carried out the analyses and wrote the first draft of the manuscript. P-YZ, YL, S-QL, Y-QZ, and M-DW carried out the patient's recruitment, clinical sample collection, and testing. TW, Z-JZ, G-BT, and XL contributed to detection and analysis of VOCs. P-YZ, Z-MC, and YL contributed to the collection of data from the electronic medical records. All authors contributed to data acquisition, data analysis, or data interpretation and reviewed and approved the final version of the manuscript.

## Funding

This work was funded by the open fund of State Key Laboratory of Respiratory Diseases (SKLRD-OP-201913); The independent fund of State Key Laboratory of Respiratory Diseases (SKLRD-Z-202019); and The Guangzhou Institute of Respiratory Health Open Project (2019GIRHZ06).

## Conflict of Interest

G-BT was employed by company Guangzhou Hexin Instrument Co., Ltd. The remaining authors declare that the research was conducted in the absence of any commercial or financial relationships that could be construed as a potential conflict of interest.

## Publisher's Note

All claims expressed in this article are solely those of the authors and do not necessarily represent those of their affiliated organizations, or those of the publisher, the editors and the reviewers. Any product that may be evaluated in this article, or claim that may be made by its manufacturer, is not guaranteed or endorsed by the publisher.

## Supplementary Material

Supplementary Figure 1 Flow chart of the enrolled participants.

Supplementary Table 1 Clinical characteristics of patients in case group and control group.
