# Supplementary material for: Exhaled Volatile Organic Compounds for Identifying Patients With Chronic Pulmonary Aspergillosis
Source: Front Med (Lausanne). 2021 Sep 23;8:720119. doi: 10.3389/fmed.2021.720119 (PMC8495266; doi:10.3389/fmed.2021.720119)
Supplement: Supplementary file 1 [file Data_Sheet_2.PDF]

Collection method:

The breath gas of the subjects was collected at 07:00 every morning, and the breath was started after rinsing with water. Before sampling, the subjects took three deep breaths by nose and mouth, and then took a deep breath through the nose. The lips were tightly closed and the breath was held for 10 seconds. After that, a small breath of gas was exhaled to discharge the air in the upper respiratory tract cavity. The disposable nozzle and connecting pipe were connected with the valve, and the blowing mouth was held until the Tedlar Bag(TDC Inc, Japan Fig.1a) was full (about 1 l volume), and the collection was repeated for three times. For the subjects who could not exhale completely through the mouth, nasal clamp was used to reduce the exhaled gas from the nasal cavity.

NOTES:

a. In order to eliminate the interference of toothpaste and oral odor, the subjects were required not to brush their teeth, and they were required to gargle with water three times before the test.

b. Sit still for 30 minutes to avoid the increase of isoprene concentration in breath due to strenuous exercise.

c. On the day before collection, the subjects were required to fast after 22:00 and not to drink drinks other than water. Excluding diet would change the content of VOCs such as acetone in breath.

d. As smoking can significantly increase the content of acetonitrile in exhaled breath, drinking will cause the content of ethanol in exhaled breath to rise in a short time. In order to avoid these interference, the subjects were forbidden to smoke and drink on the day before sampling.

e. In order to avoid the influence of Capsicum on the content of 2-pentylfuran in exhaled breath, the subjects were fasted before blowing.

f. Three parallel samples were collected for breath samples of all subjects as background and parallel samples for comparison.

g. The fixed ventilation room is selected as the gas production environment.

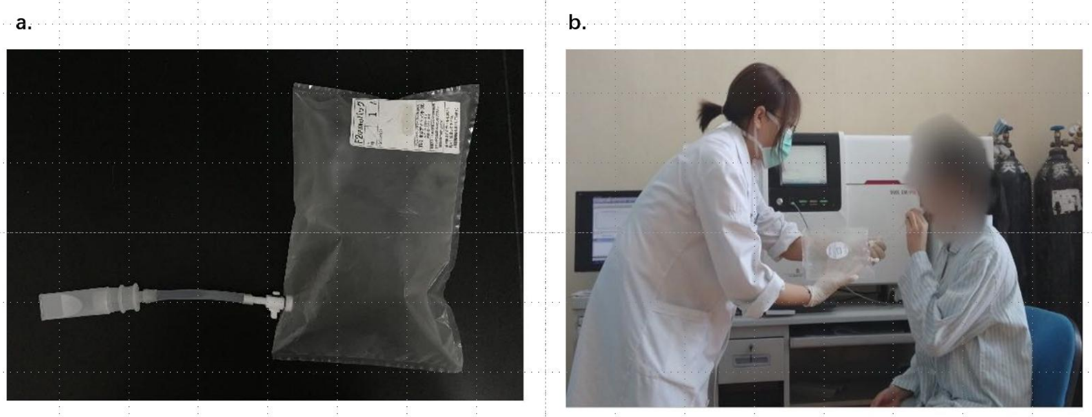

Figure1 a. Tedlar Bag b. The process of collecting breath samples
