# Supplementary material for: Exhaled Volatile Organic Compounds for Identifying Patients With Chronic Pulmonary Aspergillosis
Source: Front Med (Lausanne). 2021 Sep 23;8:720119. doi: 10.3389/fmed.2021.720119 (PMC8495266; doi:10.3389/fmed.2021.720119)
Supplement: Supplementary Table 1 — Clinical characteristics of patients in case group and control group. [file Data_Sheet_1.PDF]

Supplementary Table 1. Clinical characteristics of patients in case group and control group

Characteristics	Group PA （n=48）	Group CAP （n=32）	P value
Underlying diseases			
COPD（n,%）	15（28.30%）	1（3.12%）	0.045*
Bronchiectasis（n,%）	20（37.73%）	2（6.24%）	0.034*
Asthma（n,%）	1（1.86%）	0	0.78
Old Healed Tuberculosis（n,%）	13（24.52%）	1（3.12%）	0.37*
History of pulmonary surgery（n,%）	18（33.96%）	1（3.12%）	0.000**
Clinical symptoms			
Fever（n,%）	22（41.50%）	26（81.25%）	0.033*
Cough（n,%）	45（84.90%）	24（75%）	0.73
Expectoration（n,%）	43（81.13%）	15（46.87%）	0.051
Hemoptysis（n,%）	36（67.92%）	0	0.000**
Dyspnea	19（35.84%）	3（9.36%）	0.047*
Chest pain（n,%）	5（9.43%）	1（3.12%）	0.80
Weight loss（n,%）	26（49.05%）	2（6.24）	0.035*
Laboratory findings			
White blood cell count (10^9/L)	6.28±3.85	9.37±3.40	0.012*
Neutrophils count (10^9/L)	5.90±3.28	7.64±2.56	0.019*
Lymphocyte count (10^9/L)	1.50±0.84	1.88±0.82	0.062
Hemoglobin (g/L)	110.88±35.77	120.03±20.76	0.045*
Platelet (10^9/L)	246.95±119.84	230.63±153.83	0.089
Procalcitonin (Rise,%)	6（11.32%）	22（68.75%）	0.003**
C-reactive protein (Rise,%)	23（43.39%）	25（78.12%）	0.084
ESR (Rise,%)	21（39.62%）	17（53.12%）	0.17
Imaging			
Cavitary lesion (n,%)	35（66.03%）	0	0.000**

Aspergilloma (n,%)	26（49.05%）	0	0.000**
Tubercle (n,%)	13（24.52%）	3（9.36%）	0.012*
Patch (n,%)	9（16.98%）	14（43.75%）	0.004**
Ground-glass opacity (n,%)	4（7.54%）	8（25%）	0.013*

Note: *: $P < 0.05$ means statistical difference; * *: $P < 0.01$ means significant statistical difference
